# A Comprehensive Review of the Rehydration of Instant Powders: Mechanisms, Influencing Factors, and Improvement Strategies

**DOI:** 10.3390/foods14162883

**Published:** 2025-08-20

**Authors:** Hedong Jiang, Nanhai Zhang, Liuming Xie, Gonglong Li, Lihua Chen, Zhenggen Liao

**Affiliations:** 1 Key Laboratory of Modern Preparation of TCM, Jiangxi University of Chinese Medicine, Nanchang 330004, China; 20201042@jxutcm.edu.cn (H.J.); ligonglong@jxutcm.edu.cn (G.L.); 2 School of Pharmacy, Jiangxi University of Chinese Medicine, Nanchang 330004, China; znh597851323@163.com (N.Z.); liumingxie@email.ncu.edu.cn (L.X.); 3 Jiangxi Provincial Key Laboratory of Effective Material Basis of TCM (2024SSY07102), Jiangxi University of Chinese Medicine, Nanchang 330004, China

**Keywords:** instant powder, rehydration, limiting factor, chemical composition, improvement strategy

## Abstract

Instant powder, with its high formulation flexibility, ease of quantification, long shelf life, and convenient storage and transportation, holds a significant position in the modern food industry and has experienced rapid development. However, most powder products not optimized for instant use often encounter issues such as surface floating and clumping during rehydration. Some products remain unevenly distributed even after stirring or extending the settling time, severely impacting consumer experience. This paper systematically reviews the key factors influencing the rehydration performance of instant powders, including chemical composition, particle microstructure, and processing techniques. It also delves into corresponding improvement strategies, such as particle structure design, particle modification, and the optimization of rehydration methods. This paper aims to provide theoretical references and practical guidance for technological innovation and product development in the field of instant powders.

## 1. Introduction

Instant powder is a powdered product formed by removing most of the moisture from the raw material, designed for easy preparation. It is a common, ready-to-eat food that can be consumed directly after being rehydrated with hot water. Driven by modern fast-paced lifestyles, the instant solubility of food powders has become a core quality feature of concern to consumers. In addition to widely recognized instant coffee and milk powder, this versatile form encompasses a wide range of products, including beverage mixes, infant formula, instant soups and sauces, protein shakes and nutritional supplements, as well as fruit and vegetable powders. The key technologies in producing instant powders are dehydration and grinding. Although methods vary, the primary objective is to convert liquid or semi-liquid foods into stable dry powders, aiming to reduce moisture content and volume, extend shelf life, and achieve rapid rehydration [1].

Rehydration is defined as the ability to rapidly and completely restore fluid levels, and it is a key quality indicator for determining whether instant powders can meet “ready-to-eat” requirements. Its essence lies in the series of complex physicochemical changes that occur within the powder as sugars, water-soluble and non-water-soluble proteins, starch, and other functional components that interact with the water medium, ultimately forming a multi-phase dispersed system comprising solutions, suspensions, and emulsions [2]. However, the diversity of sources of instant powder determines that it has different chemical compositions and different spatial distributions. Food powders that have not undergone instantization often exhibit poor rehydration performance, clumping, layering, and difficulty in achieving uniformity. These issues not only compromise product uniformity and result in a gritty texture in the solution but also significantly reduce consumer sensory experience. Improving the rehydration performance of instant powders has been a key focus of research for many scholars [3,4].

Previous studies have primarily focused on the rehydration characteristics of high-protein dairy powder, providing detailed descriptions of measurement methods and their applicability in online analysis [5]. Other reviews have explored the structure, processing techniques, and common challenges of powdered foods, including various aspects of the rehydration process [6]. Although these studies have provided valuable insights into specific types of powders, systematic research on the diversity of factors such as chemical composition, particle microstructure, preparation conditions, and their interactions remains insufficient. Therefore, this review will conduct an in-depth analysis of potential rehydration mechanisms and propose corresponding improvement strategies, with a particular emphasis on innovative approaches to particle structure design, molecular structure modification, and preparation method optimization. By integrating fundamental scientific principles with practical application strategies, this review aims to provide a robust theoretical framework and practical guidance for the rapidly developing instant powder sector, driving technological innovation and product development.

## 2. Rehydration Mechanism of Instant Powders

**Figure 1:**
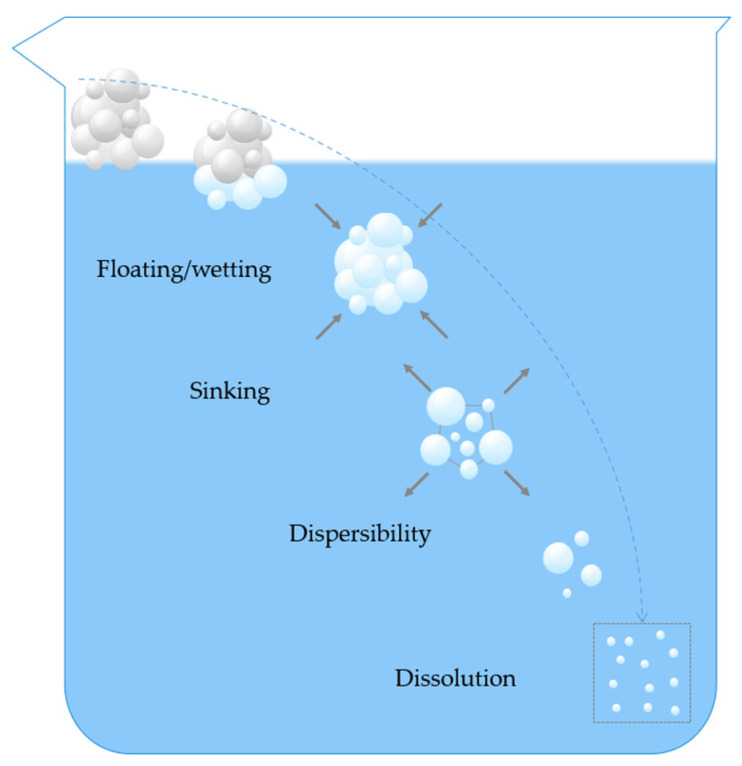
Diagram of the rehydration process of instant food powder.

The rehydration process of instant powders is typically broken down into four interrelated and partially overlapping stages: wetting, sinking, dispersibility, and dissolution [7,8] (Figure 1).

Wetting refers to the initial stage of contact with a liquid and is the first step in rehydration. The liquid penetrates into the voids between powder particles and wets their surfaces until the entire powder is wetted. Essentially, the new solid–liquid interface replaces the original solid–gas interface. The extent of wetting completion is closely related to the surface properties of the powder and the interactions between the solid and water molecules. The surface of powder particles acts as the “first barrier” during rehydration, and its properties directly determine the efficiency of subsequent rehydration stages [9]. From the perspective of surface chemical composition, the wettability of a solid surface primarily depends on the type and arrangement of its outermost atoms or functional groups, as well as the resulting surface energy. Solids bonded by strong interactions such as covalent bonds, ionic bonds, or metallic bonds have high surface energy and are easily wetted by liquids; in contrast, molecular solids bonded by van der Waals forces or hydrogen bonds have lower surface energy, resulting in relatively poor wettability. Therefore, compared to organic polymer materials, which are mainly held together by weak intermolecular forces, many inorganic solids that are bound by strong chemical bonds are usually more easily wettable [10]. However, for some hydrophilic polymer powders, such as arrowroot powder, when they come into contact with hot water, their surfaces rapidly absorb water and swell, forming a gel layer that prevents further water penetration, effectively encapsulating the internal dry powder and preventing further water from entering, resulting in undesirable clumping [11].

Sinking refers to the process by which wetted powder particles overcome the surface tension of a liquid and sink to the bottom of the liquid surface. This process is primarily influenced by the liquid surface tension, particle density, and porosity. The higher surface tension of the liquid hinders particle sedimentation, causing them to float. When particle density is low, their sedimentation rate in the fluid is slower, which also impedes the sedimentation process. The effect of porosity on sinking is a result of the combined effect of two opposing actions: on one hand, high porosity means that more gas can be adsorbed between particles, which is unfavorable for sinking; on the other hand, high porosity facilitates better transport of liquid into the interior of the particles, thereby promoting sinking [12]. Furthermore, while reducing solvent surface tension helps overcome particle floating, it may also weaken the capillary penetration effect, making it difficult for liquid to enter the interior of the powder bed via capillary action, thus delaying the sinking process [13]. For soluble instant powders, their contact angle gradually decreases during dissolution, which enhances their “sinkability” [4].

Dispersibility is a critical step in the rehydration of instant powders and refers to the uniform distribution of powder particles in a liquid medium. This process involves the breakdown of agglomerates and aggregates into their constituent powder particles. Rapid and complete dispersion is essential to ensure optimal sensory characteristics and promote the rapid dissolution of instant powder products. Dissolution is the final stage of the rehydration process, involving the decomposition of powder particle structures and the release and dissolution of molecules within the particle matrix into the liquid to form a uniform solution. However, when components are insoluble, expansion may occur instead of dissolution.

The above four sub-processes may occur simultaneously and interact with each other in the actual rehydration process. Difficulties in rehydration may arise from obstacles in any one of these stages or from problems existing in multiple stages simultaneously.

## 3. Factors Influencing the Rehydration Properties of Instant Powders

### 3.1. Chemical Composition

Food instant powders can be customized through flexible formulation design using a variety of food ingredients to meet the physiological, nutritional, and taste preferences of different populations. From a chemical perspective, their composition primarily includes various nutrients such as proteins, lipids, carbohydrates, vitamins, trace elements, and minerals. In products with different applications, the proportions of each nutrient vary significantly, directly affecting their rehydration performance. Based on public perception and food retailer classifications, Ren et al. (2024) [4] selected nine of the most common instant powder products. Products with different formulations exhibit considerable differences in the proportions of carbohydrates, fats, proteins, and additives.

#### 3.1.1. Fat

Increases in fat content in powders generally have two adverse effects on the rehydration performance. First, fat molecules are characterized by weak polarity or non-polarity and exhibit high hydrophobicity. They tend to accumulate on the surface of powder particles, forming an oil film that hinders effective contact between powder and water, thereby reducing wettability and dispersibility. Baldelli et al. (2022) [14] indicated that the dispersion time of milk powder increases with increasing fat content. A high proportion of fat in instant powder formulations is frequently associated with impaired rehydration performance [15,16,17]. It is worth noting that the extent to which fat affects dispersibility also depends on whether it is located inside or on the surface of the particles, a point that will be discussed in detail in Section 3.2.1. Secondly, most fats in natural foods bind to other biomolecules through hydrogen bonds, hydrophobic interactions, electrostatic interactions, van der Waals forces, and other mechanisms. This binding leads to a decrease in solubility, which is caused by factors such as enhanced hydrophobic interactions, altered crystal structures, increased molecular or intermolecular interactions, and enhanced thermal stability. For example, the interaction of fat with the amylose portion of starch and the hydrophobic residues of proteins typically increases their hydrophobicity and alters the structure of biomacromolecules, thereby reducing the water solubility of the resulting complex [18,19]. Cervantes-Ramírez et al. (2020) [20] demonstrated that covering the surface layer of corn starch granules with a fatty acid layer in the form of flakes via low-shear extrusion significantly reduced the solubility of the resulting binary complex.

#### 3.1.2. Protein

Proteins are widely used in infant formula and instant powders, primarily to enhance the nutritional value of the products [21]. Proteins in instant powders typically come from dairy proteins and plant proteins. Among these, dairy proteins can be further subdivided based on composition and processing into whole milk powder, skim milk powder, milk protein isolate, milk protein concentrate, whey protein isolate, whey protein concentrate, casein, and caseinates [22]. Casein accounts for a high proportion of animal proteins and typically exists in the form of micelles, which have a relatively complex structure. Casein micelles are formed by the association of various casein molecules with minerals such as calcium and phosphorus. Their surface charges and hydration layers are relatively stable. During rehydration, water molecules must overcome a certain energy barrier to enter the micelle interior and fully bind with protein molecules, resulting in a slower rehydration rate. Furthermore, animal protein molecules possess numerous hydrogen bonds, hydrophobic interactions, and disulfide bonds, leading to the formation of a compact structure in the dried state, which hinders water molecule penetration and thus affects rehydration [23]. McSweeney et al. (2020) [24] found that when the protein content in MPC > 65% (*w*/*w*), the dispersibility and solubility of the powder significantly decreased, while reducing the protein content in the powder improved its rehydration performance.

Plant proteins are increasingly being used in instant powdered foods, with soybeans being the primary source. The composition and solubility characteristics of different plant proteins are critical in determining their effectiveness in instant products. Legume proteins, such as those found in soybeans and peas, are primarily composed of water-soluble albumin and salt-soluble globulin. Due to their inherent good solubility, legume proteins exhibit excellent dispersibility and rapid rehydration properties in instant products [25]. In contrast, cereal and nut plant proteins, such as those from wheat, corn, or various nuts, primarily contain prolamins and glutelins, which have lower water solubility. This leads to difficulties in dissolution, easy clumping, or precipitation when directly applied to instant foods [26]. Therefore, processing techniques such as enzymatic hydrolysis, physical modification, or chemical modification are often required to improve their solubility and other functional properties to better suit the requirements of instant products [27]. In summary, different protein sources, due to their inherent protein composition and solubility characteristics, vary in their application methods and required processing in instant foods, and understanding these differences helps optimize product formulation and processing.

#### 3.1.3. Carbohydrates

Carbohydrates are classified into monosaccharides, disaccharides, oligosaccharides, and polysaccharides. Monosaccharides, disaccharides, and oligosaccharides, due to their smaller molecular structures, are highly soluble in water, thereby reducing powder clumping and promoting instant solubility [24,28]. Polysaccharides are formed by multiple monosaccharide molecules connected via glycosidic bonds, featuring long molecular chains, high polymerization, and numerous hydroxyl groups on their branches. These hydroxyl groups readily form intermolecular hydrogen bonds. This unique structure results in poor solubility in water [29]. Common insoluble polysaccharides include certain plant cellulose and starch. Plant cellulose possesses high water-holding capacity and swelling properties, promotes dispersibility, and is often added to instant powders to enhance taste, texture, and promote gut health. Starch is a common carbohydrate component in instant powders; when exposed to hot water, starch granules absorb water and swell, releasing amylose and increasing viscosity, as seen in instant porridge and soups. To overcome some limitations of natural starch, such as insolubility in cold water, poor paste stability, retrogradation, and inconvenience of thermal gelatinization, researchers have focused on a novel cold-water-soluble starch—granular cold-water-soluble starch (GCWS). GCWS is a modified starch that retains its complete granular structure, enabling it to disperse easily in cold water without clumping and exhibiting good swelling and thickening properties. The water solubility of GCWS starch is significantly higher than that of pregelatinized (PG) starch. Li et al. (2014) [30] obtained GCWS and PG buckwheat starches through physical modification, and their water solubilities at 50 °C were 93.33% and 4.35%, respectively, showing a significant difference.

#### 3.1.4. Inorganic Salts

Inorganic salts primarily influence the rehydration performance of instant powders in three ways. First, inorganic salts have excellent hygroscopic properties, which enhance the wettability of the powder and thereby promote rehydration. Second, inorganic salts interact with biomolecules through complex mechanisms that improve rehydration. These interactions include regulating protein charges through electrostatic shielding; influencing protein conformation through ion-specific hydrophobic interactions; disrupting protein micelle structures; and even forming ionic bridges between negatively charged protein molecules [31]. Takano et al. (2020) [32] indicated that adding a small amount of inorganic salt can effectively counteract the gelling effect of hydrophilic polymers, thereby improving dispersibility. It is noteworthy that salt concentration has a dual effect on protein solubility. Low concentrations of neutral salts can promote protein solubility in water (“salting-in”), primarily by electrostatically shielding the protein surface charges and reducing the tendency of protein molecules to aggregate. Conversely, high concentrations of neutral salts decrease protein solubility (“salting-out”), which is mainly due to mechanisms such as salt ions competing with water molecules, leading to dehydration of the protein’s hydration shell and an increase in solution surface tension [33,34]. Thirdly, the addition of salt can alter the physicochemical properties and structure of proteins. Hussain et al. [35] observed that after adding NaCl to natural micellar casein (NMC) dispersions, there was an increase in irregular structures and a decrease in β-sheet structures, along with the formation of approximately 20 nm micro-micelles, which improved rehydration.

### 3.2. Microstructure

#### 3.2.1. Surface Properties

The surface properties of particles significantly affect their rehydration performance. Hydrophobics, such as lipids and proteins with exposed hydrophobic regions, significantly hinder the wetting process if they are present on the particle surface. For example, fat in dairy powder tends to migrate to the particle surface, leading to increased surface hydrophobicity. Even with very small amounts of fat, proteins, along with the limited fat, will dominate the surface, presenting a sequential arrangement of fat, protein, and lactose [36,37]. This excessive tendency for lipids and proteins to be present on the surface is independent of production conditions [38]. Furthermore, a high-protein surface may also form an interconnected network of protein micelles, creating a dense film that results in poor rehydration properties [39,40]. Conversely, the presence of hydrophilic components such as carbohydrates, minerals, and surfactants can shorten the wetting time, thereby promoting rehydration [41]. For instance, under similar particle size conditions, skim milk powder and whole milk powder have surface lipid contents of 23% and 87%, respectively, which results in the wetting time of whole milk powder (68 s) being significantly longer than that of skim milk powder (35 s). Burgain et al. (2017) [42] found that the rehydration performance of powders has a significant correlation with their surface atomic ratio. The surface atomic ratio effectively reflects the surface composition properties of the powder. The relationship between the surface atomic ratio and the surface composition properties is shown in Table 1 [43].

#### 3.2.2. Particle Shape

Instant powder particles exhibit distinct morphological characteristics. For example, skim milk powder particles have a wrinkled surface, while whole milk powder particles are spherical. The main reason lies in the different fat contents and their distribution and physical behavior during the drying process. Whole milk powder has a relatively high fat content. During the spray drying process, fat globules are evenly distributed through emulsification, forming a continuous fat film on the surface of the particles and a protective layer around the particles. This protective layer reduces the shrinkage stress caused by the rapid evaporation of water during the drying process, ultimately maintaining the smooth and intact spherical structure of the particles. In contrast, skimmed milk powder has an extremely low fat content, and its granules are mainly composed of proteins, lactose, and other components. During the drying process, these components undergo varying degrees of moisture loss and shrinkage, resulting in uneven collapse and indentation inside and on the surface of the particles, leading to a wrinkled structure [44,45]. The roughness, morphology, and shape of powder particles are closely related to their wetting behavior [46]. Ding et al. (2020) [47] indicate that powder rehydration properties are correlated with particle shape, in addition to particle size and chemical composition. This includes shape factors such as circularity, convexity, and area. In general, physical models suggest that more spherical particles with smoother surfaces tend to exhibit better flowability and rehydration properties because they have lower friction and cohesive forces, which promote water penetration and particle dispersion. However, in complex food systems, this generalization is not absolute because factors such as chemical composition often play a more significant role. For instance, despite their lower sphericity, skim milk powder particles usually demonstrate superior rehydration properties compared to whole milk powder particles. This is primarily because the fat layer on whole milk powder’s surface hinders water penetration, whereas skim milk powder’s composition is more conducive to water interaction. Therefore, the impact of particle shape on rehydration must be considered in the context of a product’s hydrophobic components and processing aids. Gaiani et al. (2011) [48] found that morphological descriptors such as size, sphericity, and convexity are closely related to the wettability, solubility, and dispersibility of milk powder and successfully developed predictive models based on these shape descriptors, confirming that size and shape descriptors are key factors influencing the rehydration properties of milk powder. Ding et al. (2021) [49] established a model for predicting the dispersibility of instant whole milk powder using the empirical cumulative distribution function values of shape factors. Ding et al. (2023) [50] quantified the surface roughness of milk powder by constructing 3D models using 3D digital photogrammetry and Reality Capture software, followed by contour slicing analysis and deviation frequency analysis of the 3D models. It is worth noting that despite these studies providing new tools for a deeper understanding of the effect of surface shape on rehydration, reliable prediction of wettability remains elusive.

#### 3.2.3. Particle Size

A reduction in particle size leads to an increase in the specific surface area and surface energy, thereby increasing the effective contact area with water molecules. Within a certain range, this typically improves dispersibility and solubility and enhances the release rate of active ingredients [51,52]. Camacho et al. (2022) [53] demonstrated a significant negative correlation between the mean particle size of orange powder and wetting time, meaning that smaller particle sizes resulted in shorter wetting times. However, the advantages of small particle size powders are not absolute. When the particle size is smaller than a certain threshold, the cohesive force between the particles increases and the gaps between them decrease. During the wetting process, the surface tension at the gas–liquid interface causes partially wetted particles to move closer together. This further reduces the gaps between particles, thereby accelerating the moisture absorption rate on the particle surface. After absorbing a certain amount of moisture, a “protective layer” or gel forms on the powder’s surface, enveloping the entire particle. This obstructs the penetration of water molecules into the interior of the particle, resulting in “wet-on-the-outside, dry-on-the-inside” agglomerates. This phenomenon reduces the moisture penetration rate under capillary action, which has an adverse effect on the powder’s overall rehydration process [54,55].

#### 3.2.4. Molecular Aggregation State

The molecular aggregation state, amorphous state, and crystalline linear state inside the instant powder have different influences on the rehydration performance of the product. Amorphous instant powders exhibit enhanced dissolution properties relative to crystalline structures, a phenomenon attributable to disparities in molecular arrangement. Amorphous powders, due to their disordered molecular arrangement, typically exhibit higher free volume and molecular mobility. This phenomenon facilitates the penetration and interaction of water molecules with the powder particles, thereby accelerating the rates of wetting and dissolution. However, amorphous materials possess a greater capacity for moisture absorption, a property that can result in the formation of caking and, consequently, hinder the rehydration process. In contrast, crystalline powders exhibit tightly ordered and stable molecular arrangements, leading to reduced hygroscopicity and caking tendency. Nevertheless, their dissolution rate is generally slower, as water molecules need to overcome stronger intermolecular forces to break down the crystalline structure [56]. Therefore, controlling the proportion of amorphous and crystalline states in powders through drying processes (such as spray drying, freeze-drying) and storage conditions is a crucial strategy for optimizing the rehydration performance of instant powders. For instance, an appropriate amount of amorphous components can facilitate rapid initial dissolution, while stable crystalline components help maintain product storage stability and prevent caking, thus striking a balance between quick rehydration and good storage performance.

### 3.3. Processing Technology

#### 3.3.1. Drying

The drying process mainly affects the rehydration properties of instant powder by altering three factors: the microstructure of the powder, its surface properties, and the molecular aggregation state [57]. High-temperature drying, such as conventional hot-air drying and certain spray drying conditions, due to rapid evaporation and high heat input, can readily lead to the rapid formation of a dense “shell” on the surface of powder particles. For instance, casein forming a barrier on the surface of milk powder ultimately results in reduced rehydration properties [58]. Similarly, impurities like proteins, lipids, or mucilage in starch processing can create a ‘film’ on particle surfaces [59], severely hindering water ingress and particle dissolution. Furthermore, elevated temperatures can induce molecular denaturation, resulting in a denser microstructure within spray-dried particles. These particles, typically spherical, exhibit weak interactions with water [60,61]. Hot-air drying, due to its high temperature, tends to cause powder particles to shrink and harden, which reduces porosity and surface area, consequently leading to relatively poor rehydration properties [62]. Conversely, low-temperature drying techniques, such as freeze-drying, or combined drying technologies incorporating vacuum, microwave, or infrared radiation, remove moisture more gently. These methods are advantageous as they maximize the retention of the powder’s porous structure, increase its effective specific surface area, and preserve a higher proportion of amorphous components. Amorphous structures generally exhibit greater water absorption capacity, which promotes rapid water penetration into the particle interior. This significantly enhances the powder’s wetting, dispersion, and dissolution capabilities [63]. Table 2 summarizes the common drying techniques, highlighting their advantages, disadvantages, and the typical properties of the resulting powders. However, even with low-temperature or combined drying, the precise control of parameters is essential to prevent localized surface hardening or undesirable structural formations that may arise from excessively rapid drying rates.

#### 3.3.2. Pulverization

Pulverization significantly affects the rehydration performance of powder by adjusting its particle size and crystallinity [69,70,71]. Huang et al. (2021) [72] demonstrated that after ultrafine grinding of common buckwheat starch, its relative crystallinity decreased while its solubility significantly increased. However, precisely controlling the degree of crushing is a necessary condition for maintaining the integrity of the material. Insufficient pulverization may fail to achieve the desired improvement in powder properties, while excessive pulverization can be counterproductive, exacerbating the tendency for inter-particle agglomeration, which significantly impacts powder dispersibility and rehydration. Savlak et al. (2016) [73] found a negative correlation (r^2^ = −0.75) between particle size and wettability after ball milling raw banana flour; particles smaller than 212 μm exhibited the lowest wettability (83.40 s), whereas other larger particles showed very high wettability (1.31~1.44 s). Consequently, systematic experimental studies are imperative to ascertain the appropriate particle size range for various types of instant powders, balancing various performance indicators to achieve the best application effect.

#### 3.3.3. Storage

Elevated temperatures and humidity during storage have been identified as primary contributing factors to powder caking, with mechanisms primarily categorized into amorphous caking and moisture-induced caking. Amorphous caking is a mechanism exclusive to amorphous powders, primarily driven by enhanced molecular mobility. At ambient temperature or when exposed to moisture, the powder’s temperature or water activity may exceed its glass transition temperature. Consequently, the powder undergoes a transition from a rigid glassy state to a more mobile rubbery state. This leads to a sharp decrease in viscosity and a significant increase in molecular diffusivity, consequently making the particle surfaces sticky and causing them to adhere to each other, forming lumps [74,75,76]. The following amorphous instant powders are particularly prone to solidification during storage: powdered vegetables, yeast and meat extracts, hydrolyzed fish protein, meat powder, hydrolyzed vegetable protein, maltodextrins and syrups, organic acids used as food additives, spray-dried milk powders, brown sugar, and instant powders containing large amounts of the aforementioned ingredients [77].

Moisture-induced caking (also known as liquid bridge caking) is a universal mechanism that can occur in all powders containing soluble components, triggered by moisture absorption [78]. The process commences with the dissolution of soluble components on the particle surfaces due to the presence of moisture. This results in the formation of liquid bridges at specific points of contact between the particles. These liquid bridges exert strong cohesive forces through capillary action, causing particles to adhere. Subsequently, the process of moisture evaporation results in the recrystallization of dissolved substances, thereby forming solid bridges that serve to permanently bind the particles together. This phenomenon is known to lead to irreversible caking. In practical storage settings, these two mechanisms may occur independently or synergistically, collectively contributing to powder caking [76]. Therefore, it is imperative to exercise precise control over the storage temperature and humidity levels in order to ensure the integrity of instant powders.

## 4. Improvement Strategy

### 4.1. Particle Structure Design

#### 4.1.1. Adding Auxiliary Materials

The addition of excipients has been demonstrated to be an effective means of enhancing the rehydration properties of instant food powders. Excipients that are frequently utilized include maltodextrin, sucrose, lactose, inorganic salts, and other hydrophilic components. These excipients can increase powder porosity, which in turn promotes water molecule migration by increasing the specific surface area, thereby accelerating powder rehydration [79]. Furthermore, the incorporation of specific excipients such as porous starch and leavening agents can further enhance the powder’s porosity. On the other hand, certain excipients can improve rehydration by modifying the powder’s surface properties or inhibiting undesirable reactions. For instance, hydrophilic sodium caseinate can significantly boost the rehydration capacity of milk powder [80]; similarly, adding whey protein can effectively prevent casein from improper folding during the drying process, thereby improving the rehydration process of milk powder [81]. Ben Abdelaziz et al. (2014) [82] observed that the addition of sugar, particularly that with a large size, improved the rehydration of cocoa powder. However, the dispersibility of cocoa powder showed a non-linear relationship with sugar content, which might also be attributed to other properties of the sugar (e.g., particle size, type). Therefore, the selection and dosage of excipients require further optimization based on specific product characteristics.

#### 4.1.2. Control of Particle Size, Porosity, and Density

Particle size, porosity, and density are crucial indicators for evaluating powder dispersion quality [83]. The “top-down” and “bottom-up” production approaches allow for the reduction and enlargement of particle size, respectively, through processes such as pulverization (or homogenization) and agglomeration, thereby enabling the adjustment of instant powder rehydration properties [84,85]. The reported particle size range varies for different types of instant powders (Table 3). For instance, Xu et al. (2024) [86] obtained ultrafine powders with improved dispersibility and wettability through air-jet pulverization. Conversely, Lee et al. (2023) [87] demonstrated that enlarging the particle size of pregelatinized starch through agglomeration technology enhances solubility. Different drying technologies impact powder porosity. Powders prepared by freeze-drying, spray freeze-drying, and foam-mat drying exhibit higher porosity than those produced by spray drying. Supercritical drying can yield nanoporous, interconnected structures. Therefore, the selection of drying technology must consider the nature of the food matrix, desired final product characteristics, cost, and processing time. Regarding density, a higher powder density allows particles to more easily overcome buoyancy and sink rapidly in liquid [88]. Good sinkability is the primary step in the instant powder rehydration process, ensuring that powder particles quickly enter the liquid interior. However, density and porosity exhibit a mutually constraining relationship in instant powder rehydration. Solely pursuing high density may compromise water permeability, while exclusively aiming for high porosity might negatively affect sinkability. In summary, the precise control of particle size, optimization of drying processes to regulate porosity, and rational design of powder density are key strategies for enhancing the rehydration performance of instant powders.

#### 4.1.3. Powder Surface Modification

The surface modification of powders is a crucial strategy for enhancing the rehydration properties of instant powders, primarily by altering the interaction between particles and water. Firstly, removing hydrophobic surface components is a direct and effective method. For example, selectively removing free fat from the surface can significantly reduce the powder’s hydrophobicity, thereby improving its wettability [93]. Secondly, hydrophilic coating or the introduction of hydrophilic groups can enhance the surface hydrophilicity of particles. Coating particles with hydrophilic substances or surfactants can reduce the contact angle and improve wettability. Angelopoulou et al. (2021) [94] enhanced the surface hydrophilicity of whole milk powder through lactose coating. Ji et al. (2017) [95] and Hailu et al. (2023) [96] demonstrated that lecithin coating improved the wettability and solubility of whey protein isolate (WPI) and skim milk powder (SMP). Emerging technologies like cold plasma treatment, by interacting active oxygen and nitrogen species with the particle surface, can etch the surface, introduce polar groups, and partially unfold proteins, significantly improving powder wettability [3]. Furthermore, microencapsulation technology offers a strategy to shield hydrophobic components within a “shell” structure, thereby imparting good dispersibility, water solubility, and stability to the powder [97]. In summary, through these diverse surface modification methods, the hydrophilic/hydrophobic balance and interfacial behavior of instant powders can be effectively controlled, leading to a comprehensive optimization of their rehydration performance.

#### 4.1.4. Nanocomplex

The nanoparticle delivery system is an effective tool for transferring insoluble components and improving the rehydration performance of instant powders. These systems use natural biomacromolecules as base materials, which are modified biologically or chemically to form various forms [98]. Their core advantage lies in their ability to enhance the solubility and bioavailability of functional ingredients, serving as excellent carriers for poorly soluble components. For instance, Xu et al. (2020) [99] prepared composite nanoparticle curcumin, casein, and soy polysaccharide; similarly, Liu et al. (2022) [100] synthesized zein-curcumin-tea saponin nanoparticles, which significantly enhanced the rehydration of curcumin. In addition, porous starch, a modified starch distinguished by its distinctive microporous structure, exhibits considerable potential at the nanoscale. It can effectively load various poorly water-soluble functional ingredients and significantly enhance the swelling volume, solubility, and water absorption capacity of starch [101]. Liu et al. (2022) [102] synthesized novel chestnut porous starch nanoparticles, which exhibited significantly higher solubility compared to native starch. Wu et al. (2021) [103] loaded silymarin as nanoparticles into porous starch using the liquid antisolvent precipitation method, markedly improving its solubility. Nanoparticle delivery systems and porous starch-based approaches enhance the rehydration performance of poorly soluble components in instant powders by optimizing the material’s structure and interfacial properties at the nanoscale. This enables the effective integration of diverse functional ingredients into instant food matrices while ensuring rapid and efficient rehydration.

### 4.2. Particle Modification

#### 4.2.1. Physical Modification

Physical modification precisely adjusts the microstructure of powders through various techniques. Commonly used techniques include ball milling, extrusion, annealing, heat treatment, ultrasonication, high-pressure homogenization, microwave processing, and cold plasma [11]. These technologies can be used in combination to significantly improve rehydration performance. Some examples are listed in Table 4. Ball milling and high-pressure homogenization effectively reduce powder particle size, increase specific surface area, and accelerate water penetration and diffusion [104]. Extrusion, microwave processing, and certain heat treatments can promote starch gelatinization, disrupt dense crystalline structures, and form loose, porous structures, facilitating rapid water entry into the powder interior. For proteins, these physical modification processes can induce moderate denaturation, depolymerization, or conformational unfolding, exposing more hydrophilic groups, enhancing water-binding capacity, and making them more amenable to dispersion and dissolution. Furthermore, technologies such as cold plasma have been demonstrated to precisely modify the surface properties of powder particles without significantly increasing their temperature. This results in the enhanced surface hydrophilicity or wettability of the powder particles, thereby accelerating the adsorption and diffusion of moisture [105,106]. These reorganizations and optimizations promote water entry into the powder interior and its association with the powder, improving the dissolution rate and dispersion uniformity of instant powders, leading to superior rehydration performance. Physical modification controls powder particle size, internal structure, and surface properties, effectively overcoming challenges encountered by traditional powders during rehydration.

#### 4.2.2. Chemical Modification

Chemical modification enhances the interaction between powder particles and water by altering the surface properties and functional groups within the molecular structure of instant powders. Table 5 lists some methods. For starch-based components, chemical modification can control their gelatinization behavior to improve rehydration and reduce caking. For instance, Zhang et al. (2022) [107] prepared kudzu root powder-fatty acid complexes, which effectively reduced the gelatinization rate of kudzu root powder, thereby preventing caking caused by powder particles being encapsulated by a gel network before sufficient water absorption. Furthermore, acetylation and acid hydrolysis can lower the gelatinization temperature of starch, making it more readily absorb water at lower temperatures; conversely, cross-linking and oxidation increase the gelatinization temperature, enhancing the structural stability of starch granules, which is particularly important in scenarios requiring control over the gelatinization rate to avoid rapid formation of viscous gels [118]. For protein and lipid components, chemical modification primarily improves their rehydration properties through covalent or non-covalent binding with hydrophilic groups. For example, the covalent conjugation of polysaccharides with proteins or polysaccharides with fatty acids can significantly enhance the solubility and dispersibility of these components at the molecular level [119]. A typical application is the glycation of proteins via the Maillard reaction, which not only increases the denaturation temperature threshold of proteins during high-temperature drying, effectively preventing a decrease in solubility due to high-temperature denaturation, but also significantly improves their rehydration properties [120]. Panthi et al. (2021) [121] found that modulating casein micelle interactions with glycomacropeptide can significantly improve the rehydration properties of high-protein milk powders.

#### 4.2.3. Enzymatic Modification

Enzyme modification involves the controlled degradation or modification of starch, protein, and fat in powder by specific enzymes to optimize their interaction with water molecules. The commonly used enzyme modification methods are mainly amylase, protease, and lipase (Table 6). For starch-based powders, glycosidases moderately hydrolyze starch chains to produce smaller sugar molecules and dextrins, reducing gelatinization and increasing hydration sites to improve the wettability and dispersibility of powders. For protein-based instant powders, the hydrolytic action of proteases can break down large protein molecules into smaller peptides and amino acids. This increases hydrophilicity, improves dispersibility, and reduces solution viscosity. For example, Ryan et al. (2022) [129] demonstrated improved rehydration properties of MPI (Milk Protein Isolate) by hydrolyzing it with the proteases Flavourzyme™, Neutrase™, and Protamex™ (Novozymes, Bagsvaerd, Denmark). For fat-based powders, lipase can catalyze the hydrolysis of triglycerides, generating free fatty acids and glycerol, thereby reducing the free fatty acid content on the powder surface and exposing more hydrophilic groups or matrices, which enhances the powder’s wettability and dispersibility. Additionally, the glycerol produced during the hydrolysis process, as a hydrophilic substance, may also contribute to the overall hydrophilicity. In all enzyme modification processes, strictly controlling the extent of enzymatic hydrolysis is crucial to avoid adverse effects such as excessively low viscosity, inappropriate texture changes, excessive bitterness, or flavor degradation, ensuring a balance between enhanced rehydration performance and overall product quality.

## 5. Rehydration Method Parameters

Adjusting the parameters of the rehydration method is a direct and effective strategy for improving the rehydration performance of instant powder, primarily achieved by precisely controlling two core parameters: liquid temperature and mechanical stirring.

### 5.1. Liquid Temperature

Liquid temperature significantly affects the rehydration performance of instant powders. Increasing water temperature reduces surface tension and viscosity, thereby reducing the cohesive force between water molecules, which typically accelerates their penetration and diffusion into the powder [139]. Many instant products exhibit faster dissolution rates in warm or hot water. McCarthy et al. (2014) [140] demonstrated that increasing the reconstitution temperature of MPC from 25 °C to 50 °C enhanced its solubility from 45.8% to 89.7%. Similar temperature-dependent solubility enhancement phenomena have also been observed in other protein-based powders, such as whey protein isolates and soy protein isolates, as well as certain carbohydrate-based instant beverages. Increased temperature helps break intermolecular forces, enhances molecular mobility, and thereby promotes dissolution. However, temperature control is critical, as excessively high temperatures may trigger a series of adverse reactions. For starch-based powders, high temperatures may cause rapid gelatinization, forming a viscous gel that encapsulates undissolved particles, hindering further water penetration, and ultimately leading to insoluble lumps or undercooked phenomena [108,141]. Carbohydrates with different molecular weights respond differently to temperature. Higher liquid temperatures facilitate the rapid dissolution of low-molecular-weight carbohydrates [54]. For protein-based powders, high temperatures can disrupt their natural structure. High-temperature rehydration reduces the thiol content and increases surface hydrophobicity while disrupting secondary structures (including random coils, β-sheets, and β-turns). These changes promote aggregate formation through disulfide bonds and hydrophobic interactions [142,143]. These protein aggregates may have negative effects on solubility, sensory properties, and nutritional value. For fat-based powders, such as whole milk powder or cocoa powder, appropriate warm water can help melt or disperse fats to improve wettability, but excessively high temperatures may cause fat separation or oxidation. Additionally, the ratio of amorphous to crystalline states in powders also affects their temperature response: amorphous components typically absorb water and dissolve rapidly at lower temperatures but are more sensitive to temperature and humidity and prone to caking; crystalline components, however, require higher temperatures to dissolve effectively. In conclusion, the influence of liquid temperature on the rehydration performance of instant powders is rather complex. Moderate heating can accelerate the penetration of water molecules and break the intermolecular forces, which is conducive to the dissolution of most instant powders. However, excessively high temperatures can lead to the gelatinization of starch groups, the destruction of protein structures, and the separation and oxidation of aliphatic groups. In addition, powders with different proportions of crystalline states respond differently to temperature. Therefore, while considering the dissolution efficiency, the product quality also needs to be taken into account.

### 5.2. Mechanical Stirring

Mechanical stirring promotes forced interaction between powder particles and the aqueous medium, thereby enhancing dispersion and promoting efficient rehydration kinetics. This is particularly true for powders prone to agglomeration or those with high viscosity upon initial wetting, such as many protein-based or high-fat powders. Appropriate stirring provides sufficient shear force to effectively break up agglomerates formed by powder particles in the liquid, enhancing the interfacial contact between water and powder, thereby significantly increasing wetting and dissolution rates [144]. Gaudel et al. (2023) [145] demonstrated that increasing stirring speed significantly shortened the dispersion and swelling time of couscous. Jeantet et al. (2009) [146] investigated the effects of temperature (26~30 °C) and stirring rate (400~1000 rpm) on the rehydration characteristics of casein powder. Their results show that a 4 °C increase in temperature has an impact on rehydration kinetics comparable to raising the stirring speed from 400 rpm to 800 rpm, which clearly demonstrates the significant role that stirring and temperature play in promoting the rehydration process. Although mechanical stirring is beneficial for powder rehydration, its intensity should not be maximized indiscriminately. This is because different components may exhibit significant differences in their sensitivity to stirring; for example, protein structures may be more susceptible to shear damage than resilient starch particles. Additionally, excessive stirring may introduce excessive air, leading to foam formation. Furthermore, the use of different mixing system types, such as manual mixing, magnetic stirring, rotor-stator mixers, or ultrasonic stirring, may affect rehydration efficiency. Manual stirring is a common method in household settings; magnetic stirrers are widely used in laboratories; and industrial applications typically employ more powerful methods, such as high-shear mixers, to achieve rapid and complete dispersion. Currently, the mechanisms by which different stirring methods affect the rehydration properties of instant powders with varying characteristics are not yet fully understood. Selecting an appropriate stirring method based on the powder’s characteristics warrants further investigation.

## 6. Conclusions and Outlook

This review has explored the scientific mechanism in the rehydration process of instant powder. A successful rehydration process requires a series of complex physical events, including wetting, sedimentation, dispersion, and dissolution. Analysis confirms that the rehydration behavior is determined by the inherent properties and structure of the powder particles themselves, including particle size, shape, surface chemical properties, etc. We emphasized the impact of manufacturing processes on these powder particles, especially drying and crushing, and how they affect the microstructure of the powder. External environmental factors, such as storage humidity and temperature, may cause caking. These influencing factors do not exist independently but coexist and are interrelated. Therefore, the challenge lies not only in controlling a single attribute but also in the comprehensive impact of each attribute on the entire rehydration path. To this end, multiple improvement strategies are proposed, including particle structure design, particle modification, and method optimization. The common goal of these technologies is to design particles with an ideal structure and hydrophilic surface, thereby meeting consumers’ demands for instant dissolution.

Despite these advancements, significant challenges remain. A major difficulty is the diversity of food matrices, which makes it challenging to develop a universal strategy for improving rehydration. The interactions between components in complex systems are not yet fully understood, making it difficult to reliably predict their performance. For researchers and industry, an ongoing challenge is balancing the pursuit of rapid rehydration with other critical product attributes, including sensory characteristics, nutritional integrity, storage stability, and cost. Additionally, the growing consumer demand for functional foods positions instant powders as an ideal delivery vehicle for bioactive components. For example, the addition of complex functional components such as active polysaccharides, dietary fiber, flavonoids, phenolic compounds, and probiotics may alter the powder’s rehydration performance in unpredictable ways. Studying these interactions is an important research frontier and is crucial for creating effective and appealing functional products. Therefore, future work should prioritize the use of advanced characterization techniques to detect dynamic changes occurring at the particle level during reconstitution. There is also an urgent need for greener and more efficient processing technologies that allow precise control over powder structure. Finally, future innovations should be consumer-centric, combining sensory science with technical performance metrics to develop high-quality instant products that truly meet market demands and provide consumers with an exceptional experience.

## Figures and Tables

**Table 1:** Relationship between the surface atomic ratio and surface properties of Instant powders.

Surface Properties	Surface Atomic Ratio		Instant Powder
	C/O Ratio	C/Other Carbon Bond Ratio	
Hydrophilic	0~2	0~1	Sugar, latex, xanthan gum, pectin, lactose, and agar gum
Neutral	2~4	1~2	Corn flour, instant coffee, flour, semolina, pea fiber, instant mashed potatoes, yeast, and skim milk
Hydrophobic	4~7	2~5	Cocoa, hazelnut, whole milk, whey protein, casein, and concentrated dairy products

**Table 2:** Common drying methods of instant powder.

Methods	Advantages	Disadvantages	Powder Properties	References
Spray Drying	Continuous, rapid, economical	Heat Damage, Low Porosity	Spherical, Dense, Poorly Soluble	[64]
Hot-Air Drying (Convective Drying)	Simple, low-cost	Time-Consuming, High Damage	Irregular, Hard, Poor Rehydration	[65]
Freeze-Drying (Lyophilization)	Maximum quality and nutrient retention	Expensive, Very Slow	Porous, Soluble, Optimal Quality	[66]
Vacuum Drying	Reduced heat damage, good quality	Slow, Prone to Collapse	Good Quality, Moderate Porosity	[67]
Combined Drying Technologies	Synergistic: faster and higher quality	Complex, high-cost, difficult control	Tunable properties; often engineered for high porosity and solubility	[68]

**Table 3:** Reported average particle size ranges for the ideal solubility of selected instant powders.

Instant Powder	Particle Size	References
Corn silk powder	364.4 μm	[52]
Green tea powder	100~180 μm	[89]
Oat powder	132~180 μm	[90]
Orange powder	<75 μm	[91]
Potato powder	<62 μm	[92]
Banana powder	>212 μm	[73]
Ginger stalk powder	<10 μm	[51]
SMP, SSMP, WMP	160, 180, and 220 μm	[48]

**Table 4:** Physical modification methods.

Methods	Sample	Effects	References
Ball milling	Rice starch	Water solubility index and swelling power are improved.	[104]
Extrusion and shearing	Kudzu starch	The granular structure of starch is destroyed, and the rehydration performance is improved	[107]
Heat treatment	Cassava starch	The agglomeration rate is significantly reduced from 42.2% to 2.97%, and the particle structure remains basically unaffected	[108]
Ultrasound	Oat starch	Ultrasound treatment increases amylose content, swelling power, solubility, light transmittance, water retention capacity, and lipid retention capacity	[109]
Ultrasound	Rice, corn, wheat and potato starches	Specific surface area and porosity are increased	[110]
High-pressure homogenization	Chestnut starch	Solubility, swelling power, and transparency are improved	[111]
Microwave	Sago starch	The amylose content and double helix degree are increased, the morphology of starch granules is changed, and water solubility is enhanced	[112,113]
Cold plasma treatment	Millet starch	Starch molecules are depolymerized, affecting amylose content, crystal structure, water solubility, and thermal properties. Water solubility is significantly increased by 6.7 times	[105,106]
Electrical activation	Rice protein	Large aggregates are depolymerized, particle size is reduced, specific surface area is increased, surface charge is enhanced, and the dispersibility index is increased from 1.89% to 49.56%	[114]
Cavitation jet technology	Soy protein	The cavitation effect generated by cavitation jet changes the structural composition of protein aggregates, reduces particle size, and improves solubility	[115]
High hydrostatic pressure treatments	Natural micellar casein	Porous powders with loose structures are produced, thereby significantly improving wetting, dispersion, and dissolution behaviors	[116]
Direct steam injection processing	Pea-rice protein isolate blends	High-molecular-weight aggregates are destroyed, surface hydrophobicity is reduced, disulfide bonds are formed, and the solubility of the blends is improved	[117]

**Table 5:** Chemical modification methods.

Methods	Sample	Effects	References
Acid modification (hydrochloric acid/malic acid)	Jackfruit seed starch	The amylose content of acid-modified starch slightly decreases, while its swelling power and water-binding capacity decrease, and solubility increases.	[122]
Ozonation	Corn starch	Ozonation leads to an increase in the number of carbonyl and carboxyl groups, significantly affecting gelatinization properties, gel texture, water absorption rate, and solubility index.	[123]
pH cycling	MPI	The cross-linking of casein micelles on the powder surface decreases, and surface porosity increases. With the increase in MPI content, the rehydration performance is improved.	[58]
Phosphorylation	Egg white protein powder	The electronegativity increases and surface hydrophobicity decreases. The particle size decreases while the specific surface area increases. The particles become looser and more porous. Phosphorylated proteins have higher solubility than non-phosphorylated proteins.	[124]
Succinylation	Rice protein	By derivatizing the ε-amino groups of lysine residues, the protein surface has small pits and protrusions, and solubility is enhanced.	[125]
Cross-linking, deamidation	Zein	Water solubility is enhanced.	[126]
Glycosylation and acylation	Soy protein isolate	Water solubility is enhanced, the viscosity coefficient (k) decreases, while the flow index (n) value increases.	[127,128]

**Table 6:** Enzymatic modification methods.

Methods	Sample	Effects	References
Pullulanase	Potato starch	Forms low-molecular-weight hydrolysates, with increased solubility.	[130]
4-α-glucanotransferase	Native starch	Alters amylose content, branch chain length distribution, and molecular weight distribution.	[131]
Maltogenic amylase	Kudzu starch	Reduces amylose content and molecular weight, but increases the proportion of short chains and branch chain density, decreases crystallinity, and enhances solubility.	[132]
Laccase and transglutaminase	Mixed lupin and whey protein powder	Improves protein solubility, emulsion stability, and foaming capacity of the mixture.	[133]
Alcalase, Protamex, Flavourzyme	MPC	Significantly increases solubility and degree of hydrolysis.	[134]
Alcalase	Pea, rice, hemp, and oat proteins	Significantly increases solubility and degree of hydrolysis.	[135]
Alcalase	Soy protein isolate	Destroys the structure of soy protein, changes its physicochemical properties, and improves its functional characteristics. The wetting time and dispersion time are reduced, and solubility is increased.	[136]
Enzymatic hydrolysis and cysteine modification	Zein	Increases hydrophilicity.	[137]
Alcalase, Brauzyn, Flavourzyme	Goat milk cheese protein	The hydrolysates exhibit high solubility.	[138]

## Data Availability

No new data were created or analyzed in this study. Data sharing is not applicable to this article.
